# Global–local feature fusion: a robust hybrid deep learning model for multiclass brain tumor classification with Grad-CAM++ interpretation

**DOI:** 10.3389/fgene.2026.1814786

**Published:** 2026-07-09

**Authors:** Kirti Pant, Pijush Kanti Dutta Pramanik, Shahid Mohammad Ganie, Anindita Saha, Zhongming Zhao

**Affiliations:** 1 Department of Computer Science and Engineering, Bipin Tripathi Kumaon Institute of Technology, Dwarahat, Uttarakhand, India; 2 School of Computer Applications and Technology, Galgotias University, Greater Noida, Uttar Pradesh, India; 3 Center for Precision Health, McWilliams School of Biomedical Informatics, The University of Texas Health Science Center at Houston, Houston, TX, United States; 4 Department of Health Information Management and Technology, College of Applied Medical Sciences, King Faisal University, Al-Ahsa, Saudi Arabia

**Keywords:** brain tumour, deep learning, explainable AI, Grad-CAM++, hybrid CNN–transformer architecture, Magnetic Resonance Imaging (MRI), Medical Image Analysis, multi-class tumor diagnosis

## Abstract

**Background:**

Accurate classification of brain tumors in MRI scans is critical for effective clinical decision making, yet manual assessment is labor-intensive and subject to variability. Although deep learning models, particularly CNNs and Transformers, have shown potential, each architecture alone has limitations: CNNs excel at local feature extraction but lack global context awareness, while Transformers capture global relationships but may overlook fine-grained details.

**Objectives:**

To develop a hybrid Transfer Learning–Transformer model that integrates CNN-driven local feature modeling with Transformer-based global reasoning to enhance multi-class brain tumor classification.

**Methods:**

The proposed workflow comprises three stages: (1) benchmarking standalone CNN (ResNet50, VGG19, ConvNeXtBase, EfficientNetV2B0) and Transformer models (ViT, Swin, DeiT, PoolFormer); (2) constructing two intra-family hybrids—H_TL_ (ResNet50 + ConvNeXtBase) and H_TF_ (PoolFormer + ViT); and (3) fusing them into a final hybrid model (H_F_). Two public MRI datasets (Figshare, Kaggle) were used. Models were trained, validated, and tested on the Kaggle dataset, while the Figshare dataset was used exclusively for external validation. Performance was assessed using accuracy, precision, recall, F1-score, AUC, Friedman’s aligned-rank test, Kendall’s W, Holm *post hoc* analysis, TOPSIS-based ranking, calibration analysis (Brier score), and Grad-CAM++ for interpretability.

**Results:**

The H_F_ model achieved near-perfect classification (∼99.4% on Kaggle and up to 100% on Figshare), significantly outperforming all baseline and single-architecture models. Statistical and multi-criteria analyses consistently ranked H_F_ as the top-performing model, while calibration results confirmed reliable probability estimation. Grad-CAM++ further indicated tumor-focused decision-making.

**Conclusion:**

The proposed hybrid model delivers high accuracy, strong generalizability, and reliable, interpretable predictions across MRI datasets, positioning it as a promising solution for AI-assisted brain tumor diagnosis.

## 1 Introduction

Brain tumors are among the most difficult neurological conditions to manage, impacting more than 300,000 individuals each year and contributing a substantial portion of cancer-related deaths, particularly in children and young adults (Jones, 2024). Tumor characteristics vary widely across histological types such as gliomas, meningiomas, and pituitary adenomas, each exhibiting distinct growth rates, invasiveness, and responses to treatment (Jaiswal et al., 2025). Both early and accurate diagnosis are critically important, as they directly influence surgical planning, radiotherapy contouring, chemotherapy decisions, and long-term prognosis. Magnetic Resonance Imaging (MRI) remains the preferred diagnostic modality owing to its superior soft-tissue contrast and non-ionizing nature (Pant et al., 2026). However, manual interpretation and classification of MRI scans are labor-intensive and prone to inter- and intra-observer variations, particularly in distinguishing borderline lesions or rare tumor subtypes (Jyothi and Singh, 2023).

Deep learning has transformed automated medical image analysis, with Convolutional Neural Networks (CNNs) providing state-of-the-art performance in brain tumor detection and classification (Magadza and Viriri, 2021; Chauhan et al., 2024). Transfer learning with architectures such as ResNet, VGG, and EfficientNet has become a popular strategy to overcome data scarcity by leveraging pre-trained weights from natural image datasets (Ottom et al., 2022; Ranjbarzadeh et al., 2021; Islam et al., 2022; Ronneberger et al., 2015; Allah et al., 2023). While CNNs excel at extracting local spatial features, their limited receptive fields restrict their ability to model long-range dependencies and broader tissue morphology, which is critical for detecting infiltrative gliomas or distinguishing tumours from treatment-induced changes (Hatamizadeh et al., 2022).

Transformers, originally developed for natural language processing, have recently shown great potential for medical imaging analysis, with architectures such as Vision Transformers (ViT) (Wang et al., 2023; Lan et al., 2023), DeiT (Touvron et al., 2022), Swin Transformer (Liu et al., 2021), and hybrid models (Das and Goswami, 2025). Their self-attention mechanism allows global contextual reasoning, yet these models often require large-scale annotated datasets and lack the inductive bias of convolutions, making them difficult to optimize in medical domains. Studies such as DBTrans (Zeng et al., 2023), FT-Vit (Reddy et al., 2024), ViTALT (Poornam and Angelina, 2024), ViT ensembles (Tummala et al., 2022), and MMMViT (Qiu et al., 2024) have demonstrated improvements, but challenges remain in model generalizability and robustness to distribution shifts. The Swin Transformer (Liu et al., 2021) (Ke et al., 2024) enhances ViTs for visual tasks by incorporating hierarchical feature learning via shifted windows, enabling multi-scale processing while maintaining linear complexity.

However, while Transformers capture global dependencies, they may underperform in encoding fine-grained local features essential for precise medical segmentation. To bridge the gap between local detail and global reasoning, hybrid CNN–Transformer architectures have emerged as a promising direction. These models leverage CNNs for fine-grained feature extraction and Transformers for learning global spatial dependencies. Prior works (Ghazouani et al., 2024; Zhu et al., 2023) demonstrate performance gains through hybridization, yet most studies focus on segmentation tasks rather than classification, or they do not address clinical reliability, interpretability, and cross-dataset robustness.

Despite these advances, current systems continue to encounter four enduring challenges:

- CNNs lack global contextual awareness,
- Pure Transformers require large datasets and struggle with fine structural details,
- Single-model systems underexploit complementary tumor characteristics across MRI sequences,
- Generalization remains weak when models are tested across datasets with different acquisition protocols.

To address these limitations, we design a hybrid transfer learning–Transformer model. The workflow consists of three stages—baseline evaluation, intra-family hybrid development, and final model fusion—which are discussed in detail in Section 4. The proposed model is trained and evaluated on two public datasets (Figshare and Kaggle), covering multiple tumor types, including glioma, meningioma, pituitary, as well as non-tumor cases. Performance is assessed using accuracy, precision, recall, F1-score, and the area under the curve (AUC). To strengthen reliability claims, statistical significance is assessed using Friedman’s aligned-rank test and Holm’s *post hoc* analysis. Model interpretability is examined using Grad-CAM++ heatmaps to ensure that predictions are clinically meaningful and anatomically grounded.

The main contributions of this study include:

- A novel hybrid architecture combining transfer learning-based CNNs and Transformer networks for multi-class brain tumor classification,
- A comprehensive three-stage evaluation strategy demonstrating progressive improvements from individual to hybrid and final fused models,
- Statistical validation using Friedman and Holm’s tests to verify that improvements are not due to random variation,
- Integration of Grad-CAM++ for visual interpretability, confirming that model decisions align with clinically relevant tumor regions,
- Cross-dataset validation demonstrating robustness to distribution shifts, supporting practical deployment in clinical environments.

The rest of the paper is organized as follows. Section 2 reviews recent literature. The research methodology, including datasets and data preprocessing, is described in Section 3. The architectural details of the proposed model are presented in Section 4. Section 5 provides experimental details, results, and performance analysis. An extensive statistical analysis of the model’s performance is provided in Section 6. The model interpretation using GradCAM++ is discussed in Section 7. An in-depth critical discussion, along with clinical relevance and practical applications, is presented in Section 8. Section 9 concludes the paper, highlighting the study’s limitations and future directions.

## 2 Related work

Automated brain tumor analysis has evolved from conventional CNN pipelines (Rasool et al., 2024) to transfer learning (Valverde et al., 2021), Transformers (Aamir et al., 2025), and hybrid/ensemble strategies (Das and Goswami, 2025). In addition to the accuracy, two concerns now shape the field: cross-dataset robustness and interpretability for clinical decision support. The following review synthesizes key lines of work with an eye toward their practical limits and what a hybrid transfer learning–Transformer model can add.

### 2.1 Traditional CNNs for brain tumor classification

Early CNN-based systems showed that end-to-end feature learning could outperform hand-crafted pipelines on routine MRI. Raza et al. (2024) designed an automated framework that combined careful preprocessing with CNN classification, reporting strong sensitivity and specificity for early tumor detection and thereby underscoring the feasibility of fully automated screening. To boost discriminative power under limited data, Ahamed and Sadia (2022) explored multimodal augmentation and loss-aware exchange to fuse complementary MRI sequences, while residual connections stabilized optimization and helped deeper models converge. Stathopoulos et al. (2024) systematically compared multiple CNN backbones across six MRI sequences and found that pairing the appropriate modality with the appropriate network increased accuracy to 98.6%, demonstrating that both architecture and sequence choice matter for clinical workflows.

### 2.2 Transfer learning for brain tumor classification

Transfer learning remains the workhorse in settings with modest sample sizes. Aggarwal et al. (2024) benchmarked VGG16, ResNet50, and InceptionV3, reporting that pretraining substantially increased performance on tumor subtype classification. Alanazi et al. (2022) demonstrated a two-stage strategy—first training a binary tumor/no-tumor CNN, then reusing weights for multi-class subtyping—which generalized well across scanners, reaching ∼96–97% accuracy on an external set. Several groups report high-nineties results with strong modern backbones: Vimala et al. (2023) fine-tuned EfficientNet variants (B0–B4) on the CE-MRI Figshare dataset, achieving ∼99% test accuracy with balanced precision/recall and Grad-CAM visualizations; Pravitasari et al. (2020) observed similar gains with EfficientNet-B5 on both binary and multi-class tasks; Korani et al. (2025) optimized InceptionV3/Xception while trimming fully connected layers and using optimizer search (including PSO), reaching ∼98–99% without heavy augmentation; Mathivanan et al. (Mathivanan et al., 2024) found MobileNetV3 highly competitive (∼99.7%) after targeted enhancement for class balance. Additional transfer-based efforts include DenseNet- or ResNet-centered variants with design tweaks to mitigate overfitting or vanishing gradients (Sharif et al., 2022; Ghassemi et al., 2020; Mehnatkesh et al., 2023; Sharma et al., 2023; Kumar et al., 2021), and attention-enhanced Xception models that emphasize tumor-relevant features while keeping parameters modest (Shaik and Cherukuri, 2022). Collectively, these studies confirm that transfer learning offers a reliable baseline and strong data efficiency, though it may still struggle with long-range dependencies or distribution shifts.

### 2.3 Transformers for brain tumor classification

Transformer-based architectures have gained increasing traction in brain tumor classification due to their ability to model long-range dependencies through self-attention—an area where CNNs are inherently limited. Tummala et al. (2022) demonstrated that fine-tuned ViT variants can reach ∼98% accuracy on contrast-enhanced MRI, with ensemble variants performing even better. Subsequent studies confirmed that, when properly regularized and fine-tuned, ViT models can match or outperform established CNNs such as ResNet, DenseNet, and EfficientNet (Reddy et al., 2024; Asiri et al., 2023a; Asiri et al., 2023b). Models designed to capture multi-scale information such as MMMViT (Qiu et al., 2024), which fuses modality-aware attention with hierarchical token processing, have further improved robustness across tumors of varying sizes. Architectures like DBTrans (Zeng et al., 2023) refined the original Transformer design with shifted-window attention and cross-window feature aggregation, highlighting the role of localized windowing in medical imaging.

Swin Transformer variants have been particularly impactful. Asiri et al. (2024) applied a Swin-based classification pipeline to four tumor classes (glioma, meningioma, pituitary, and non-tumor), achieving 97% accuracy and outperforming CNN, DCNN, and ViT baselines. Alam et al. (2025) advanced this further using Swin Transformer V2 with a Dual-Branch Downsampling mechanism and enhanced attention modules, achieving 98.97% accuracy on a dataset of 7,023 MRI images. Ferdous et al. (2023) addressed data scarcity by proposing LCDEiT, a linear-complexity data-efficient Transformer trained via a teacher–student strategy, reporting over 98% accuracy on Figshare and strong generalization to BraTS-21. Munaganuri et al. (2025) combined Data-efficient Image Transformers (DeiT) with Firefly Algorithm-based hyperparameter optimization to yield high accuracy, while Munaganuri et al. (2025) integrated DeiT with PCA and adaptive differential evolution (ADE), achieving 95.69% accuracy and demonstrating the utility of evolutionary feature optimization.

Despite these gains, Transformer-only architectures have limitations. They often demand larger labeled datasets, incur higher computational cost, and may miss fine-grained local details due to weak inductive bias. These constraints have driven a shift toward hybrid CNN–Transformer frameworks that can jointly capture local structural cues and global spatial context.

### 2.4 Hybrid and ensemble models for brain tumor classification

Hybrid models aim to merge the strengths of CNNs—strong local texture extraction and inductive bias—with Transformers’ ability to capture long-range dependencies. Early CNN-based hybrids, such as ResNet–VGG combinations or stacked 2D-CNN frameworks, reported moderate gains by exploiting complementary feature hierarchies, while strategies like SMOTE helped address class imbalance in multi-class MRI data (Anwar et al., 2025) (Pillai et al., 2023). Ensemble approaches, combining CNNs with LSTM or multi-branch feature extractors, improved robustness by averaging error tendencies across models and datasets (Islam et al., 2024). Attention-based dual-stream networks (e.g., InceptionResNetV2 + Xception) further refined localization of tumor regions with minimal preprocessing (Bodapati et al., 2021). Although primarily developed for segmentation, architectures combining CNN feature encoders with Swin-based attention (ELSA, Swin-UNet) (Ghazouani et al., 2024; Zhu et al., 2023; Cao et al., 2023) demonstrate that hybrid designs consistently perform well when both local structure and contextual relations are preserved.

Recent Transformer-dominant hybrids follow the same principle but with more efficient architectural integration. Bataineh et al. (2024) combined Swin Transformer with ResNet50V2, where the Transformer captures global relationships and the CNN backbone stabilizes feature extraction. This model achieved 99.9% accuracy on binary and 96.8% on multi-class MRI datasets, outperforming VGG, EfficientNet, ConvNeXt, and plain CNN variants. Pacal (2024) refined Swin using a Hybrid Shifted-Window Self-Attention and a Residual MLP head, improving training speed and memory efficiency while achieving 99.92% accuracy. Almuhaimeed et al. (2025) added AE-cGAN augmentation to a Swin-based classifier, addressing data imbalance and improving generalization, reaching up to 99.54% (Figshare) and 98.9% (Kaggle). Across these studies, a common pattern emerges: hybridization with Transformers improves contextual reasoning, CNN backbones stabilize fine-detail extraction, and synthetic augmentation boosts performance under limited or imbalanced data.

### 2.5 XAI for brain tumor classification

Interpretability methods increasingly accompany high-accuracy models to support clinical trust (Hosny and Mohammed, 2025). Grad-CAM/Grad-CAM++ and LIME are common methods for verifying that predictions attend to pathologic regions. Examples include lightweight CNNs paired with interpretable classifiers (Nahiduzzaman et al., 2025), DenseTransformer-style hybrids with attention blocks and post-hoc explanations (Panigrahi et al., 2025), and segmentation-classification pipelines where saliency maps help scrutinize decision boundaries (Abd-Elhafeez et al., 2024; Ullah et al., 2024). In classification settings, heatmaps serve as a fast plausibility check, especially useful for tumor boards and second-read workflows, providing overlays are co-registered, normalized, and archived for audit.

### 2.6 Research gap and scope of this study

Despite advancements, three gaps persist. First, CNN-only systems excel at local structure but miss global relationships necessary for complex morphology or distant context, while Transformer-only systems capture global context but may underperform on fine boundaries or require more data to stabilize. Second, many single-architecture models exhibit performance declines under domain shifts (scanner/protocol/population), limiting clinical portability. Third, while XAI is increasingly reported, few studies combine *statistically validated* performance (e.g., aligned-ranks tests with post-hoc correction), *cross-dataset robustness*, and *interpretable outputs* in a single classification pipeline.

This study targets those gaps by:

- Benchmarking strong transfer learning CNNs and canonical Transformers;
- Constructing intra-family hybrids and a final fusion that integrates both families;
- Validating on two public datasets (Figshare and Kaggle) using standard metrics;
- Testing statistical significance via Friedman’s aligned ranks and Holm’s post-hoc procedures; and
- Providing Grad-CAM++ visualizations to verify that the model’s attention aligns with clinically meaningful regions.

The result is a classification framework designed for high sensitivity, balanced precision, cross-dataset stability, and transparent decision support.

## 3 Research methodology

**FIGURE 1:**
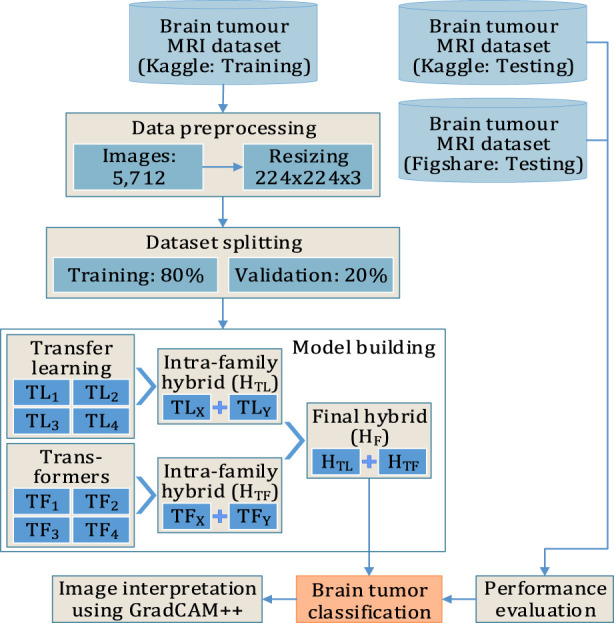
Research methodology.

Figure 1 presents the overall framework for building an effective brain tumor classification system from MRI images. The approach integrates two deep learning families, transfer learning and Transformers, to derive a robust and accurate model. In contrast to conventional hybrid CNN–Transformer architectures that rely on direct or loosely coupled feature fusion, the proposed framework adopts a staged hybridization strategy, where feature integration is performed progressively rather than in a single step.

Multiple transfer learning and Transformer models are first explored independently. Based on their initial performance, two intra-family hybrid models are constructed—one that consolidates CNN-based representations and the other that integrates Transformer-based features. This intra-family consolidation enables the alignment of homogeneous feature spaces before combining heterogeneous representations. These two hybrid branches are then fused to derive the final model, in which local spatial features (from CNNs) and global contextual representations (from Transformers) are integrated into a unified embedding space.

The framework outputs one of three tumor classes along with the normal (no tumor) class. The model’s performance is assessed using standard evaluation metrics, and Grad-CAM++ is employed to visualize prediction results. For training and testing, separate datasets are utilized, ensuring improved generalizability across varying data distributions.

### 3.1 Dataset description

This study uses two publicly available brain MRI datasets: one from Kaggle (Nickparvar, 2021) for model development and another from Figshare (Cheng, 2024) for external evaluation. The Kaggle dataset is divided into two predefined folders: Training (5,712 images) and Testing (1,311 images). From the training folder, 80% of the images were used for training and the remaining 20% for validation, while the Testing folder was used exclusively for internal evaluation.

To assess generalization, an independent dataset from Figshare was used for external testing. The original Figshare dataset comprises 2,870 MRI slices but is highly imbalanced. From this, a balanced subset of 591 images was selected using stratified random sampling to preserve class distribution (glioma: 143, meningioma: 152, pituitary: 152, no-tumor: 144). This design ensures a fair evaluation across all tumor categories while avoiding bias toward dominant classes. The choice of subset size reflects a trade-off between computational feasibility and representativeness. The objective of this experiment was not to maximize sample size but to evaluate whether a model trained on Kaggle generalizes to an independent dataset with a different distribution. In this context, maintaining class balance was prioritized over using the full dataset.

It is also important to note that while the Figshare dataset consists of contrast-enhanced T1-weighted MRI slices, the acquisition sequence for the Kaggle dataset is not explicitly specified. This variation introduces cross-domain heterogeneity, allowing evaluation of the model’s robustness under realistic clinical conditions.

Table 1 provides the class-wise distribution for the Kaggle training and validation sets, whereas Table 2 summarizes the class distribution in the internal Kaggle test set and external Figshare test subset.

**TABLE 1:** Detailed class-wise distribution of the Kaggle (training) dataset.

Splitting sets	Glioma	Meningioma	Pituitary	No_tumor	Total
Training (80%)	1056	1071	1165	1276	4568
Validation (20%)	265	268	292	319	1144

**TABLE 2:** Detailed class-wise distribution of the test sets.

Splitting sets	Glioma	Meningioma	Pituitary	No_tumor	Total
Kaggle	300	306	300	405	1311
Figshare	143	152	152	144	591

#### 3.1.1 Both the datasets comprise the following four classes

Glioma: Gliomas are the most prevalent brain tumours, originating from glial cells that support and protect neurons. They account for approximately 30% of all brain and central nervous system tumours and nearly 80% of malignant brain tumours. Gliomas may arise in various regions of the brain and are graded according to their degree of malignancy (e.g., grades I-IV). Common symptoms often include headaches, seizures, memory impariment, and behavioural or cognitive changes.

Meningioma: Meningiomas develop in the meninges, the protective layers covering the brain and spinal cord. They are usually benign and slow-growing, accounting for approximately 30% of all primary brain tumours. Although often asymptomatic initially, they can cause symptoms like headaches, vision disturbances, seizures, or neurological deficits when they press against surrounding brain tissue.

Pituitary tumour: Pituitary tumours arise in the pituitary gland, a small organ at the base of the brain that produces hormones. These tumours are typically benign (adenomas), but their location can affect hormonal balance and vision. Symptoms may include headaches, vision problems, fatigue, unexplained weight changes, and hormonal imbalances that affect growth, metabolism, or reproductive function.

No tumour: Normal brain MRI images show no evidence of tumor growth, structural abnormalities, or lesions. These scans are used as a reference to distinguish pathological brain conditions and play a crucial role in training models for accurate brain tumor classification, just as regular CT scans are used in lung cancer detection.

### 3.2 Data preprocessing

To develop a robust, reliable automated system for brain tumour classification, data preprocessing is crucial to the model-building process. Preprocessing is an essential step to eliminate distortions from MRI images and to standardize the data for practical analysis. In this study, preprocessing steps such as image resizing, normalization, and on-the-fly data augmentation were employed to enhance the quality and diversity of the dataset. These steps help in improving the model’s ability to generalize and accurately detect various types of brain tumours. The specific preprocessing techniques utilized are detailed in the subsequent sections.

The preprocessing phase includes image resizing, normalization, and on-the-fly data augmentation using Albumentations (Buslaev et al., 2020). To ensure uniformity in input data and compatibility with the employed deep learning models, all images in the dataset were resized to a standard resolution of 224 × 224 pixels. This resizing was performed using the cv2.resize function from OpenCV. On-the-fly data augmentation is performed to improve generalization of images without increasing the size of the dataset. Spatial augmentations are applied, including horizontal and vertical flips with probabilities of 0.5 each, and rotations limited to ±20 degrees with 70% probability, which helps generalize the model by varying orientations. To simulate realistic deformations in the images, the Elastic Transform is applied. Finally, images are normalized by scaling pixel values to a range.

## 4 Proposed hybrid model

**FIGURE 2:**
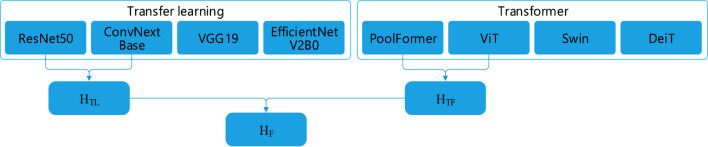
Hybridising approach adopted in this study.

This study proposes a three-stage hybrid framework (Figure 2) for brain tumor classification using MRI, combining transfer learning-based convolutional networks and Transformer architectures to leverage both local and global feature representations. The workflow begins with Phase 1, where eight baseline models are independently trained and evaluated: four CNN-based transfer learning models (ResNet50, VGG19, ConvNeXtBase, EfficientNetV2B0) and four Transformer-based models (ViT, Swin, DeiT, PoolFormer). This stage establishes the performance ceiling of individual architectures and identifies their strengths: CNNs in extracting fine-grained local features and Transformers in capturing long-range spatial dependencies.

In Phase 2, two intra-family hybrid backbones are constructed. First, the ResNet50 and ConvNeXTBase backbones are used to extract features. Then, MHCA layers are added to each model, followed by feature processing using Average2D pooling and a Flatten layer. The classifier head is developed with a fully connected layer, two SwiGLU layers with dropout for classification. The first, H_TL_, fuses ResNet50 and ConvNeXTBase to consolidate shallow, mid-level, and deep convolutional features into a unified 256-dimensional descriptor. The second hybrid, H_TF_, is developed by extracting features from the PoolFormer and ViT models, followed by adding an MHCA attention layer to PoolFormer. For ViT, CLS Token extraction is performed before FeatureHarmonizer. Afterwards, an Average Pool2D layer is added in PoolFormer, followed by flattening and the FeatureHarmonizer. Both branches receive the same preprocessed MRI input and are trained end-to-end. Each branch outputs a latent feature tensor of size (32, 256), where 32 corresponds to the batch size and 256 represents the learned embedding dimension.

Phase 3 integrates these two complementary feature spaces into the final hybrid fusion model (H_F_). The 256-dimensional feature vectors from H_TL_ and H_TF_ are concatenated to form a 512-dimensional unified feature representation of shape (32, 512). This fused vector preserves convolution-based spatial granularity while embedding Transformer-derived global structure. The joint representation is passed through a fully connected feature fusion module consisting of two linear layers: the first projects from 512 × 512 to 512 × 256, followed by GeLU activation and dropout (p = 0.5), and the second reduces from 512 × 256 to 256 × 256, again followed by GeLU and dropout (p = 0.3). This stage minimizes redundancy, enhances feature discriminability, and prevents overfitting.

**FIGURE 3:**
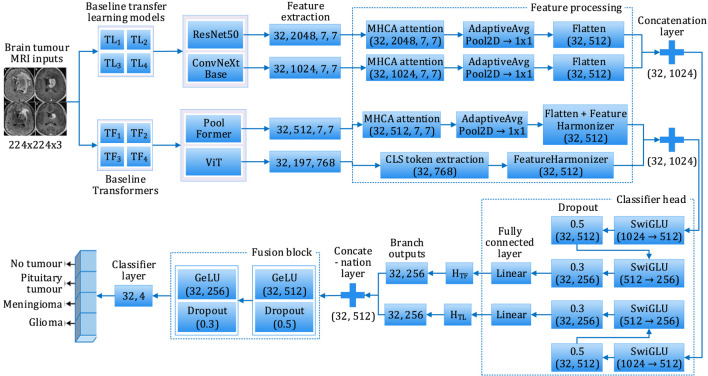
Architecture of the proposed hybrid model for brain tumor classification.

The final classification block comprises a linear layer that maps the 256-dimensional fused representation to four output logits corresponding to glioma, meningioma, pituitary tumor, and no-tumor classes. Softmax activation is applied to produce normalized class probabilities. The architecture of the complete model is illustrated in Figure 3. The model is optimized using the Adam optimizer with a learning rate of 1 × 10^-4^ and trained with a batch size of 32 over 20 epochs. Categorical cross-entropy is used as the loss function, and dropout is applied throughout the fully connected layers to improve generalization. All architectural and hyperparameter configurations are provided in Tables 3, 4. The complete algorithm for this procedure is shown in Algorithm 1.

**TABLE 3:** Description of the proposed hybrid model’s architecture.

Layer	Action/Type	Output dimension
H_TL_	HybridResNetConvNeXt (Identity)	(32,256)
H_TF_	HybridPoolFormerViT (Identity)	(32,256)
Concatenate	None	(32,512)
Fusion fc_1_	Linear(512 x 512)	(32,512)
GeLU	Activation	(32,512)
Dropout	p = 0.5	(32,512)
Fusion fc_2_	Linear(512 x 256)	(32,256)
GeLU	Activation	(32,256)
Dropout	p = 0.3	(32,256)
Classifier	Linear(256 × 4)	(32,4)

**TABLE 4:** Hyperparameter settings of the considered hybrid models.

Metric	Metric value		
​	H_F_	H_TL_	H_TF_
Batch size	32	32	32
Optimizer	Adam	Adam	Adam
Epochs	20	16	16
Learning rate	1e-4	1e-4	1e-4
Criterion	categorical_crossentropy	categorical_crossentropy	categorical_crossentropy
Early stopping	Patience = 10	5	5

Algorithm 1 **Proposed hybrid model building process.**

1. **Input:** Brain MRI dataset (MRI images)
2. **Output:** Tumor class label *C* ∈ {glioma, meningioma, pituitary, no tumor}
3. // Step 1: Dataset Loading and Preprocessing
4.  Load MRI images from the Kaggle (Training and Testing) and Figshare datasets.
5.  Apply preprocessing pipeline, including resizing, rescaling, and on-the-fly augmentation on the Kaggle (Training) dataset.
6.  Shuffle and split the Kaggle (Training) dataset: 80% training and 20% validation.
7. // Step 2: Training on Baseline Transfer Learning Models and Transformers
8. Train and evaluate Transfer learning models: ResNet50, VGG19, EfficientNetV2B0 and ConvNeXT-Base on Kaggle (training) dataset.
9.  Train and evaluate Transformers: PoolFormer, ViT, Swin Transformer and DeiT on Kaggle (Training) dataset.
10. // Step 3: Building Intra- Transfer and Transformer Hybrid Models
11.  Extract features using ResNet50 backbone and ConvNeXt backbone → apply MHCA layer at each backbone → apply adaptive average pooling to both → flatten.
12.  Concatenate flattened features to form a combined feature vector(32, 1024).
13.  Pass combined features through fully connected layers containing two SwiGLU layers and two dropout layers → output class logits for four classes → optimize with cross-entropy loss and Adam.
14.  Extract features from PoolFormer backbone → apply MHCA attention and adaptive pooling → harmonize features with normalization and GeLU activation.
15. Extract features from ViT backbone by taking the [CLS] token output → harmonize features with normalization and GeLU activation.
16. Concatenate both feature vectors → pass through SwiGLU-based fusion layers with dropout → classify with a final linear layer → output logits for four classes.
17. // Step 4: Building Proposed Hybrid Model
18. Extract 256-D features from HybridResNetConvNeXt and HybridPoolFormerViT models by replacing their classifiers with nn.Identity() → yield two (32, 256) feature vectors.
19. Concatenate these feature vectors to form a combined vector (32, 512) → input to (fusion fc1 (Linear layer: 512 x 512) → GeLU activation → Dropout(0.5)) && (fusion fc2 (Linear: 512 x 256) → GeLU activation → Dropout(0.3)).
20. Feed the resulting vector (32, 256) to classifier (Linear: 256 → num classes) to output logits for target classification.
21. // Step 5: Model Training
22. The final hybrid model is trained on Kaggle (Training) dataset.
23. // Step 6: Model Testing/Evaluation
24. The final hybrid model is tested on Kaggle (Testing) and Figshare datasets.
25. Evaluate the model using Classification reports, AUC score, ROC curve.

## 5 Experiments, results and performance analysis

This section presents the experimental details used to evaluate the proposed hybrid model for brain tumour classification. The experimental setup was developed and evaluated on Google Colab with A100 GPU. Python served as the primary programming language, utilizing the Google Colab distribution for environment and package management. A detailed summary of the hardware and software specifications used in the study is provided in Table 5. The studied models are evaluated using standard evaluation metrics—accuracy, precision, recall, F1-score, and AUC—to ensure comprehensive performance assessment.

**TABLE 5:** System’s hardware and software specifications for the experiment.

Category	Component	Description
Hardware	Google Colab environment	Cloud-based Jupyter notebook environment providing computational resources
	Processor	Intel Xeon processor (provided by Google Colab)
	Memory (RAM)	40GB or 80GB (provided by Google Colab)
	Graphics Processing Unit (GPU)	NVIDIA A100 (provided by Google Colab)
	Storage	Google Drive for storing data, code, and models
Software	Programming language	Python
	Deep learning libraries	TensorFlow, Keras, PyTorch, Timm
	Computer vision libraries	OpenCV
	Data processing & visualization	Pandas, NumPy, Matplotlib, Seaborn
	Machine learning utilities	Scikit-learn
	Data augmentation	Albumentations
	Explainable AI	GRAD-CAM++
	Other libraries	TQDM

**FIGURE 4:**
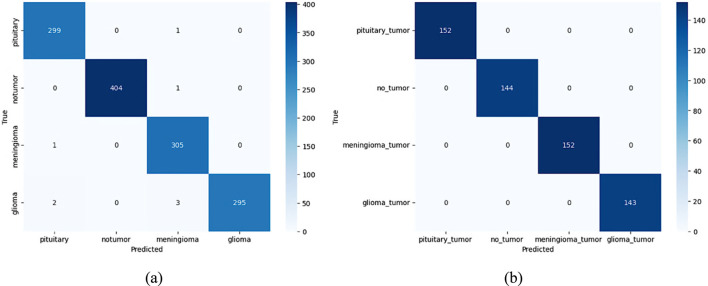
Confusion matrices of the proposed hybrid model on **(a)** Kaggle and **(b)** Figshare dataset.

The confusion matrices from both Kaggle and Figshare datasets, shown in Figure 4, indicate that the hybrid transfer-Transformer-based model (H_F_) demonstrates strong tumor segmentation capability across different tumor classes. A high concentration of values along the diagonal suggests that the model reliably distinguishes between tumor and non-tumor regions, reflecting strong true positive rates and minimal misclassification. This implies effective feature extraction from both global contextual information (via Transformers) and localized spatial patterns (via transfer learning backbones).

For tumor-positive classes, the model shows high sensitivity, meaning it correctly identifies most of the tumor pixels or slices. This is crucial in medical diagnosis, where false negatives (missed tumor regions) are clinically more problematic than false positives. The relatively lower numbers in the off-diagonal entries for these classes indicate that the model seldom confuses different tumor types or mislabels them as healthy tissue.

The false positive occurrences, although minimal, likely correspond to regions with ambiguous textures or edema-like characteristics. These errors are more evident around boundary regions of the tumors, a known challenge in MRI-based segmentation. However, the low rate of misclassification in non-tumor regions indicates strong model precision and robustness against over-segmentation.

The performance metrics of the proposed hybrid model along with other considered models, are given in Table 6. Across both datasets, the final hybrid (H_F_ = H_TL_ + H_TF_) is the top performer. On Figshare, it hits 100% on all five metrics—accuracy, precision, recall, F1-score, and AUC—leaving no daylight for alternatives. On Kaggle, H_F_ remains at the top tier with accuracy/precision/recall/F1 ≈ 99.35–99.39 and AUC ≈ 99.99, essentially tying H_TL_ and edging every Transformer-only or CNN-only baseline. That pattern—ceiling results on Figshare and resilient high-nines on Kaggle—signals genuine generalization rather than dataset overfitting.

**TABLE 6:** Comparing the proposed model’s performance with other considered models.

Dataset	Model	Accuracy	Precision	Recall	F1	AUC
Kaggle	ResNet50	97.86	97.79	97.71	97.75	97.92
	ConvNextBase	99.31	99.26	99.28	99.27	99.95
	VGG19	98.47	98.41	98.36	98.38	99.94
	EfficientNetV2B0	98.86	98.84	98.77	98.79	99.97
	PoolFormer	98.86	98.82	98.78	98.80	99.95
	ViT	98.70	98.63	98.66	98.64	99.94
	Swin	97.56	97.56	97.36	97.38	99.95
	DeiT	98.55	98.46	98.45	98.45	99.93
	H_TL_	99.39	99.35	99.36	99.35	99.97
	H_TF_	99.24	99.21	99.19	99.2	99.98
	H_F_	99.39	99.35	99.36	99.35	99.99
Figshare	ResNet50	98.98	99.05	98.95	98.98	98.98
	ConvNextBase	99.83	99.84	99.83	99.83	100
	VGG19	98.48	98.57	98.46	98.49	99.95
	EfficientNetV2B0	98.82	98.87	98.78	98.81	99.99
	PoolFormer	99.49	99.5	99.5	99.5	100
	ViT	99.66	99.68	99.65	99.66	100
	Swin	98.82	98.9	98.78	98.81	99.99
	DeiT	99.15	99.19	99.14	99.16	99.95
	H_TL_	99.32	99.34	99.33	99.33	99.98
	H_TF_	99.66	99.66	99.67	99.67	100
	H_F_	100	100	100	100	100

Among single models, ConvNeXtBase and ViT are consistently strong, but neither matches the hybrids. H_TL_ (ResNet50 + ConvNeXtBase) delivers a clear uplift over its parts on both datasets; H_TF_ (PoolFormer + ViT) is the best Transformer-side hybrid, nearly matching the overall leaders on Figshare (F1 ≈ 99.67) and staying in the 99.2–99.24 Accuracy band on Kaggle. This says the CNN–CNN fusion captures complementary local texture and boundary detail, while the Transformer–Transformer fusion stabilizes global context; H_F_ then stacks those complementary error profiles and wins by reducing both boundary misses (FN) and spurious edge activations (FP).

**FIGURE 5:**
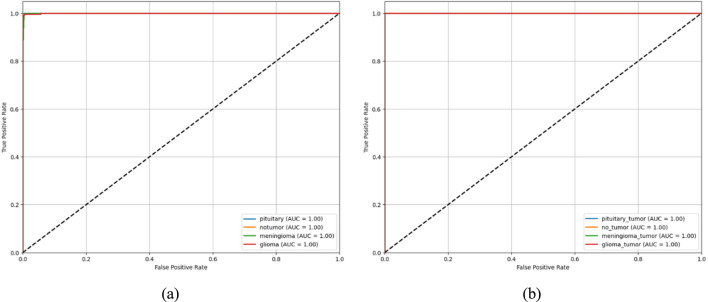
ROC curves of the proposed hybrid model on **(a)** Kaggle and **(b)** Figshare dataset.

The ROC curves for the final hybrid model (H_F_) on both the Kaggle and Figshare datasets, shown in Figure 5, reveal a consistently high discriminative ability between tumor and non-tumor regions. In both cases, the curves closely follow the top-left boundary of the plot, indicating that the model achieves an excellent balance between sensitivity and specificity across a range of threshold values. This behaviour suggests that the model is not overly dependent on a particular threshold and maintains stable decision-making even in the face of uncertainty.

On the Kaggle dataset, although the AUC is marginally lower, it remains very close to 1.0, demonstrating strong generalization despite differences in image quality, scanning parameters, and annotation style. The slight deviation from the ideal ROC path reflects minor variations in prediction confidence but does not indicate a meaningful decline in diagnostic reliability. For the Figshare dataset, the ROC curve shows a near-perfect step-like pattern, with an AUC approaching 1.0. This suggests that the model makes very few misclassifications, particularly minimizing false negatives—which is critical in medical imaging, where missing tumor pixels is far more consequential than occasional over-segmentation.

Table 7 compares the performance of our proposed hybrid model (across both datasets) with the state of the art using various parameters and performance metrics. The comparative results demonstrate that the proposed hybrid model offers performance that is competitive with, and in several aspects superior to, existing state-of-the-art brain tumor classification techniques. Most previous studies report accuracies in the range of 97%–99% across datasets such as Kaggle, BraTS, Figshare, and BR35H, often using CNN-based architectures, Transformer models, or ensemble frameworks. However, only a few achieve consistent high performance across multiple datasets while also incorporating statistical validation and interpretability—two elements critical for clinical adoption.

**TABLE 7:** Comparing the proposed hybrid model with the state-of-the-art.

Ref.	Dataset used	No of images	No. of classes	Splitting ratio (train: [validation]: test)	Model with best accuracy	Accuracy (%)	Precision (%)	Recall (%)	F1-score (%)	AUC (%)	XAI	Statistical analysis
Farhan et al. (2025)	BraTS2020 dataset	494	3	70:15:15	Ensemble dual-modality U-Net	98.36	98.80	98.03	97.73	-	√	X
Hasan et al. (2023)	BrainTumorInSight	1625	2	80:10:10	ResNet50	98.03	85.02	86.74	85.80	-	√	X
Zeineldin et al. (2022)	BraTS2019 dataset	335	2	-	ResNet-50	98.62	-	-	-	-	√	X
Mala et al. (2025)	BraTS2020 dataset	369	4	80:10:10	Multi-scale attention U-Net	99.39	99.42	99.26	99.34	-	√	X
Nazira et al. (2024)	BR35H dataset	3060	2	90:08:02	Customized CNN	98.67	98.50	98.50	98.50	98.65	√	X
Iftikhar et al. (2025)	Kaggle dataset	7023	4	80:10:10	XAI-based CNN model	99.21	99.22	99.18	99.20	-	√	X
	NeuroMRI dataset	3264	4	-		94.72	94.70	95.10	94.63	-	√	X
Lakshmi et al. (2025)	BT-MRI dataset from Kaggle	5500	4	-	XAISS-BMLBT	97.75	95.56	95.42	95.48	98.00	√	X
Shanto et al. (2025)	Kaggle dataset	7023	4	-	Hybrid CNN-Transformer	99.00	100	100	99.00	-	√	X
Kumar et al. (2023)	Collection of ACRIN-DSC-MR-Brain (ACRIN 6677/RTOG 0625), CPTAC-GBM, and ACRIN-FMISO-Brain (ACRIN 6684)	1572	2	-	Novel CNN	98.85	97.50	96.38	97.34	-	X	X
Asiri et al. (2024)	Figshare dataset	2870	4	85:15	Swin Transformer	97.00	96.00	96.25	96.00	-	X	X
Alam et al. (2025)	Kaggle dataset	7023	4	80:10:10	Improved Swin Transformer V2	98.97	98.75	98.51	98.63	-	X	X
Ferdous et al. (2023)	Figshare dataset	233	3	-	LCDEiT	98.11	97.86	97.84	97.85	0.99	X	X
	BraTS2021 dataset	2040	4	-		93.69	93.68	93.68	93.68	0.97		
Srinivas et al. (2025)	Kaggle dataset	7023	4	80:20	DeiT and Firefly algorithm	99.70	99.60	99.80	99.50	-	X	X
Bataineh et al. (2024)	BraTS2021 dataset	3160	4	80:10:10	SwT+Resnet50V2	96.80	-	97.00	97.00	-	X	√
	BrH35 dataset	3000				99.90	-	99.90	99.90	-		
Almuhaimeed et al. (2025)	Kaggle dataset	3000	4	80:20	AE + Swin Transformer	99.54	99.54	99.54	99.54	99.80	X	X
	Figshare dataset	3064				98.90	98.90	98.90	98.90	99.80	​	​
Pacal (2024)	Kaggle dataset	7023	4	80:20	Swin Transformer	99.92	99.92	99.92	99.92	-	X	X
This paper	Kaggle dataset	7023	4	80:20	Proposed hybrid model	99.39	99.35	99.36	99.35	99.99	√	√
	Figshare dataset	591	4	00:00:100		100	100	100	100	100	√	√

On the Kaggle dataset, the proposed hybrid model achieves 99.39% accuracy, 99.35% precision, 99.36% recall, 99.35% F1-score, and an AUC being close to 1.0, placing it among the top-performing methods. This performance is comparable to high-scoring methods like Pacal’s Swin Transformer (99.92%), Srinivas et al. using DeiT with Firefly optimization (99.70%), and Almuhaimeed et al. (99.54%). However, unlike these models, our approach demonstrates two additional strengths: (i) it generalizes effectively to a second dataset (Figshare), where it achieves 100% across all evaluation metrics, and (ii) it includes both Grad-CAM++–based interpretability and statistical validation (Friedman and Holm tests), which are absent in many related works.

While models such as Swin Transformer-based approaches and CNN–Transformer hybrids have reported high accuracy, they often lack external validation or remain dataset-specific. For example, models trained solely on Kaggle or BraTS datasets frequently do not evaluate cross-dataset generalization. In contrast, the proposed model maintains perfect scores on the external Figshare dataset, demonstrating robustness against variations in acquisition protocol, tumor morphology, and scanner differences.

Furthermore, only a subset of prior work incorporates XAI tools (e.g., Grad-CAM, LIME), and even fewer provide statistical significance testing. The inclusion of both in this study offers stronger evidence of reliability and clinical usability. The ability to visualize decision regions and confirm performance through hypothesis testing addresses two major barriers to deployment: transparency and reproducibility.

Overall, while accuracies among recent methods are approaching saturation, the strength of the proposed hybrid model lies in its consistent cross-dataset performance, integration of complementary CNN–Transformer features, statistically validated superiority over baselines, and clinically meaningful interpretability. These characteristics make it more suitable for real-world diagnostic workflows than single-architecture or dataset-specific models.

## 6 Statistical analysis

To assess the statistical significance of the proposed hybrid model, we utilized the nonparametric Friedman’s aligned ranks test (Hodges et al., 2012) for each performance metric. Subsequently, we performed *post hoc* pairwise comparisons using the Holm correction method (Holm, 1979), maintaining a significance threshold of 0.05.

### 6.1 Friedman’s aligned ranks test

To assess whether the performance differences observed between the proposed hybrid model and other baseline and hybrid models (per metric) were statistically significant, Friedman’s aligned ranks test was employed. This nonparametric test is designed for comparing multiple algorithms evaluated on identical tasks, thus accommodating the repeated-measures structure typical in such settings. The aligned-ranks variant improves sensitivity by removing block effects prior to ranking, enabling a fair, distribution-free comparison when models exhibit closely competing performance.

Table 8 reports the overall Friedman’s statistics and p-values together with the decision on H0 for the two datasets while the ranks of each model for both datasets are tabulated in Table 9. At the 0.05 significance level, the Friedman aligned-ranks test rejects the null hypothesis of equal performance across models for both datasets (Kaggle: statistic = 43.03, p ≈ 0; Figshare: statistic = 42.21, p = 1e-5), confirming that the observed differences are statistically significant.

**TABLE 8:** Friedman aligned ranks test statistics.

Dataset	Statistic	p-value	Result
Kaggle	43.03389	0.00000	H0 is rejected
Figshare	42.20878	0.00001	H0 is rejected

**TABLE 9:** Model-wise ranking of Friedman aligned test.

Rank	Kaggle dataset		Figshare dataset	
	Rank score	Algorithm	Rank score	Algorithm
1	49	H_F_	49.4	H_F_
2	48.5	H_TL_	46.2	ConvNextBase
3	43.2	ConvNextBase	41.9	H_TF_
4	40.8	H_TF_	40.9	ViT
5	27.5	PoolFormer	36.6	PoolFormer
6	27.4	EfficientNetV2B0	24.4	H_TL_
7	21.9	ViT	20.9	DeiT
8	18.4	DeiT	13.6	Swin Transformer
9	15.5	VGG19	13.4	EfficientNetV2B0
10	9.6	Swin Transformer	12.6	ResNet50
11	6.2	ResNet50	8.1	VGG19

To further quantify the consistency of these rankings, Kendall’s coefficient of concordance (W) was computed. The obtained values—0.8620 for Kaggle and 0.8460 for Figshare—indicate strong agreement among model rankings across evaluation metrics. This high level of concordance reinforces that the observed performance hierarchy is not driven by metric-specific variability but reflects a stable and reliable ordering of models.

With significance and ranking consistency established, the ranking tables become meaningful indicators of relative merit. On both datasets, the final hybrid (H_F_) occupies the top position (Kaggle = 49.0; Figshare = 49.4), demonstrating stable first-place performance despite variations in acquisition protocols and tumor morphology. The pattern beneath the leader is instructive. On Figshare, ConvNeXtBase ranks second and H_TF_ third, while H_TL_ drops to sixth; on Kaggle, H_TL_ rises to second, ConvNeXtBase to third, and H_TF_ places fourth. This crossover suggests that individual CNNs and Transformer hybrids remain sensitive to dataset-specific characteristics, whereas H_F_—the fusion of H_TL_ and H_TF_—retains its superiority across conditions. In effect, the fusion model mitigates the error tendencies of its components and maintains stability under distributional shifts, a known limitation of single-family architectures in medical imaging. The lower-ranked models, including Swin, VGG19, and ResNet50, consistently cluster toward the bottom, while EfficientNetV2B0 remains mid-tier, highlighting the limitations of conventional or less adaptive architectures under varying data conditions.

### 6.2 Post hoc analysis

To further explore differences in pairwise performance, a *post hoc* analysis was conducted using the Holm step-down method. This approach was chosen because it strikes a balance between controlling the family-wise error rate and maintaining adequate sensitivity to identify true differences. This makes Holm particularly appropriate for studies comparing algorithms.

We compared the proposed hybrid model against other baselines and the hybrid models across all the five performance metrics for both datasets. The results (Table 10) confirms that the final hybrid (H_F_) is statistically distinguishable from most single-family baselines on both datasets. Specifically, the null hypothesis (no difference) is rejected for comparisons against VGG19, ResNet50, Swin Transformer, and DeiT across Figshare and Kaggle (adjusted p ranging from ∼2.4 × 10^−4^ to 1.8 × 10^−2^), establishing a clear and consistent advantage for H_F_ over these base CNN and Transformer models.

**TABLE 10:** Post hoc test of the proposed hybrid model with the other models for each metric on both datasets.

Kaggle dataset				Figshare dataset			
Comparison	Statistic	Adjusted p-value	Result	Comparison	Statistic	Adjusted p-value	Result
H_F_ vs ResNet50	4.22405	0.00024	H0 is rejected	H_F_ vs VGG19	4.07601	0.00046	H0 is rejected
H_F_ vs Swin Transformer	3.88849	0.00091	H0 is rejected	H_F_ vs ResNet50	3.63189	0.00253	H0 is rejected
H_F_ vs VGG19	3.30621	0.00757	H0 is rejected	H_F_ vs EfficientNetV2B0	3.55294	0.00305	H0 is rejected
H_F_ vs DeiT	3.02000	0.01769	H0 is rejected	H_F_ vs Swin Transformer	3.53320	0.00305	H0 is rejected
H_F_ vs ViT	2.67457	0.04489	H0 is rejected	H_F_ vs DeiT	2.81274	0.02947	H0 is rejected
H_F_ vs EfficientNetV2B0	2.13176	0.16513	H0 is accepted	H_F_ vs H_TL_	2.46732	0.06806	H0 is accepted
H_F_ vs PoolFormer	2.12189	0.16513	H0 is accepted	H_F_ vs PoolFormer	1.26327	0.82597	H0 is accepted
H_F_ vs H_TF_	0.80928	1.00000	H0 is accepted	H_F_ vs ViT	0.83889	1.00000	H0 is accepted
H_F_ vs ConvNextBase	0.57242	1.00000	H0 is accepted	H_F_ vs H_TF_	0.74020	1.00000	H0 is accepted
H_F_ vs H_TL_	0.04935	1.00000	H0 is accepted	H_F_ vs ConvNextBase	0.31582	1.00000	H0 is accepted

Two models show dataset-dependent behavior. Against ViT, H_F_ is not statistically different on Figshare (H0 accepted; adjusted p = 1.000) but becomes significantly better on Kaggle (H0 rejected; adjusted p ≈ 0.0449). Compared with EfficientNetV2B0, H_F_ shows no difference on Figshare (H0 accepted; adjusted p ≈ 0.165) but a significant gap on Kaggle (H0 rejected; adjusted p ≈ 0.0031). These asymmetries track the harder distribution of the Kaggle set and reinforce that H_F_’s edge grows under domain shift.

As expected for an ensemble that fuses the strengths of both worlds, H_F_ is statistically not much distinguishable from the other hybrids—H_TL_ and H_TF_—on both datasets (H0 accepted; adjusted p ≥ 0.068). The same lack of significance holds against the strongest single backbones (ConvNeXtBase and PoolFormer) after Holm correction. Practically, this means H_F_ remains the top-ranked method in aggregate testing, but its margins over the best contenders are small enough that multiple-comparison control renders them non-significant at the pairwise level—precisely the pattern you want from a well-calibrated, conservative posthoc analysis.

### 6.3 TOPSIS-based multi-criteria ranking

Beyond statistical comparisons, a multi-criteria decision-making (MCDM) analysis was conducted to evaluate model performance when multiple metrics are considered jointly. To this end, the Technique for Order Preference by Similarity to Ideal Solution (TOPSIS) was employed to generate a composite ranking of all models across the test datasets.

TOPSIS determines the most favorable model by quantifying its relative proximity to an ideal solution. Given a set of alternatives evaluated across multiple criteria, each model is assessed based on its Euclidean distance from the ideal best (maximum metric values) and the ideal worst (minimum metric values). The relative closeness is computed as:

$$C_{i}^{*}=\frac{S_{i}^{-}}{S_{i}^{+}+S_{i}^{-}}$$

where $S_{i}^{+}$ and $S_{i}^{-}$ represent the distances from the ideal best and worst solutions, respectively. The resulting score $C_{i}^{*}\in \left[0,1\right]$ , with higher values indicating better overall performance.

**FIGURE 6:**
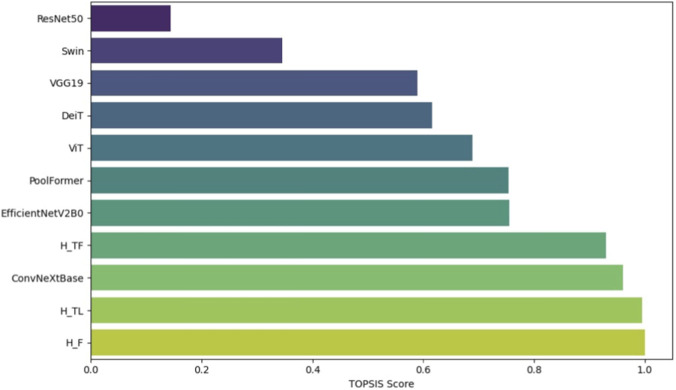
TOPSIS-based multi-criteria ranking of models on the Kaggle dataset.

**FIGURE 7:**
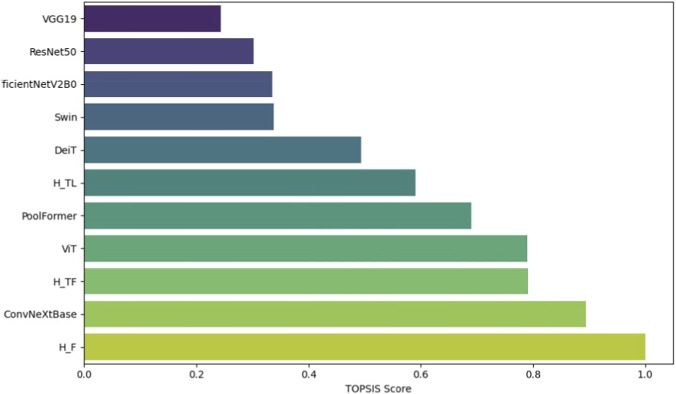
TOPSIS-based multi-criteria ranking of models on the Figshare dataset.

The TOPSIS rankings for Kaggle and Figshare datasets (Figures 6, 7) show a consistent pattern: H_F_ attains the highest score (≈1.00) across both datasets, confirming its superiority under a unified multi-metric evaluation. On the Kaggle dataset, H_F_ slightly outperforms H_TL_ and ConvNeXtBase, followed by H_TF_, while all single CNN and Transformer models rank lower. Notably, traditional architectures such as ResNet50 and Swin fall significantly behind, indicating limited competitiveness in a multi-metric setting.

On the Figshare dataset, H_F_ again ranks first with a clear margin, followed by ConvNeXtBase and H_TF_, while H_TL_ shows relatively lower positioning compared to Kaggle. Transformer models (ViT, PoolFormer) perform moderately well, but still remain inferior to hybrid configurations. The ranking pattern across both datasets consistently reflects a performance hierarchy: single models < intra-family hybrids < cross-family fusion (H_F_).

### 6.4 Critical difference analysis

**FIGURE 8:**
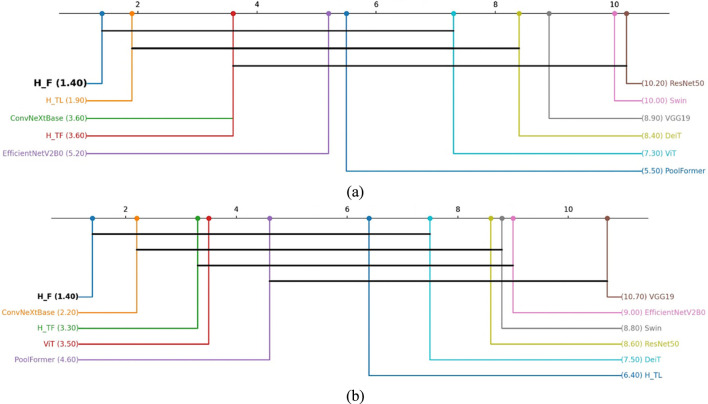
Critical difference diagram of model rankings on the **(a)** Kaggle dataset and **(b)** Figshare dataset.

Critical difference (CD) diagrams are used to illustrate how models are ranked relative to one another and to indicate whether statistically significant differences, as determined by the Friedman and Holm tests, exist. In this study, the CD diagrams are constructed based on the TOPSIS-derived model rankings, thereby reflecting a multi-criteria evaluation across all performance metrics. Figure 8 presents the CD diagrams for the Kaggle and Figshare datasets. Each diagram provides a rank-based comparative assessment of all models, where lower average rank indicates better performance, and statistically indistinguishable groups are connected by horizontal bars.

Across both datasets, H_F_ consistently achieves the lowest average rank (∼1.40), clearly positioning it as the top-performing model. In the Kaggle dataset, H_F_ is closely followed by H_TL_ (∼1.90), while all remaining models—including ConvNeXtBase, H_TF_, ViT, and others—are ranked noticeably higher. Importantly, the CD bars show that H_F_ is not statistically tied with most baseline models, indicating that its superiority is not marginal but statistically meaningful. Even strong individual models such as ViT and ConvNeXtBase fall into higher-rank groups, separated from H_F_ by the critical difference threshold.

A similar pattern is observed on the Figshare dataset, where H_F_ again secures the best rank (∼1.40). While ConvNeXtBase (∼2.20) and H_TF_ (∼3.30) appear relatively competitive, they remain outside the closest rank position. The grouping structure further indicates that H_F_ forms a distinct top cluster, with limited or no statistical overlap with weaker-performing models such as VGG19, EfficientNetV2B0, and ResNet50.

Overall, the CD diagrams reinforce three key observations:

- Consistency: H_F_ maintains the top rank across both datasets, demonstrating robustness.
- Statistical separation: The gap between H_F_ and most baselines exceeds the critical difference, confirming non-trivial performance gains.
- Hierarchical improvement: Intra-family hybrids (H_TL_, H_TF_) rank above individual models, while the final hybrid (H_F_) dominates all, validating the staged hybridization strategy.

### 6.5 Calibration curve

Calibration curves assess how closely predicted probabilities align with true outcome frequencies, providing insight into the reliability of model confidence beyond raw accuracy. In this study, they are used to verify whether H_F_ produces trustworthy probabilistic outputs, where curves closer to the diagonal indicate better calibration.

**FIGURE 9:**
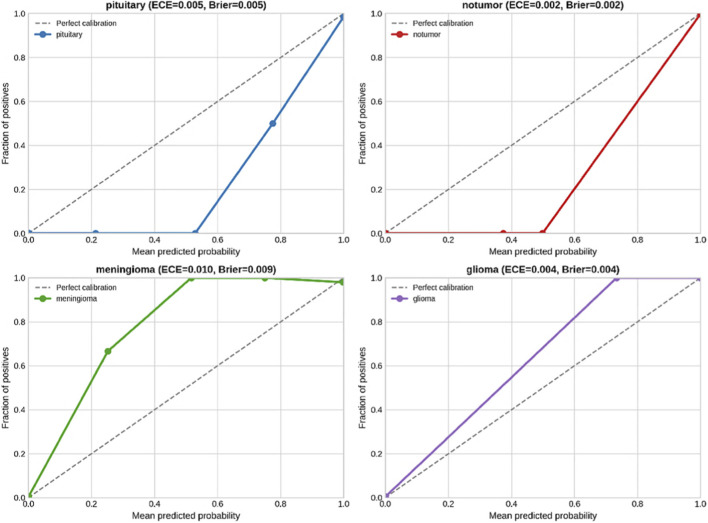
Multiclass calibration curves with Brier score analysis on the Kaggle test set (H_F_ model).

**FIGURE 10:**
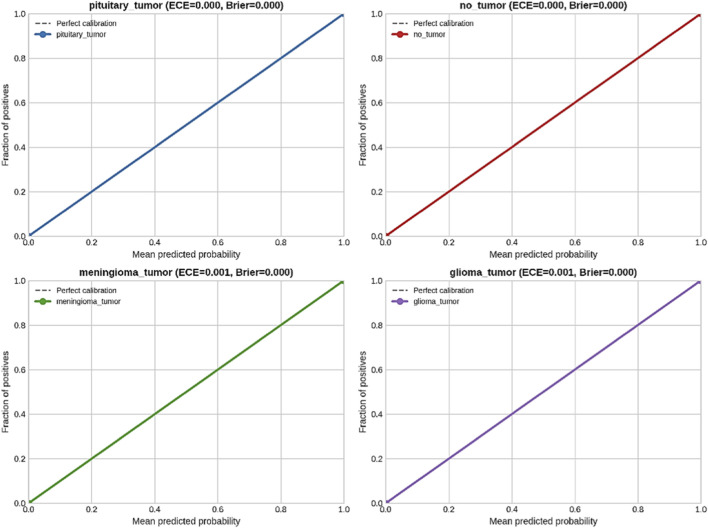
Multiclass calibration curves with Brier score analysis on the Figshare test set (H_F_ model).

As shown in Figures 9, 10, across both Kaggle and Figshare datasets, H_F_ demonstrates consistently strong calibration across all tumor classes. On the Kaggle dataset, the curves closely follow the ideal diagonal, as evidenced by low expected calibration error (ECE) values (≈0.0019–0.0096) and Brier scores (≈0.0017–0.0091). Minor deviations, particularly for meningioma, suggest slight overconfidence in certain probability ranges, yet overall calibration remains robust. The overall log loss (0.0468) and Brier score (0.0196) further confirm the stability of uncertainty estimation in the presence of realistic data variability. On the Figshare dataset, calibration is nearly perfect, with curves almost exactly overlapping the diagonal. This is reflected in negligible ECE values (≈0–0.001) and extremely low Brier scores (10^−6^–10^−4^), along with an overall Brier score of 0.0002.

The calibration curves suggest that H_F_ not only achieves high predictive accuracy but also maintains well-calibrated confidence estimates, with Kaggle reflecting realistic operating conditions and Figshare representing near-ideal performance.

## 7 Computational efficiency analysis

In addition to predictive performance, computational efficiency was examined to assess the practical feasibility of the proposed framework. The analysis, summarized in Table 11, considers model complexity (in terms of parameter count), training time, and inference behavior, providing a balanced view of deployment cost.

**TABLE 11:** Computational efficiency comparison of models in terms of parameters, training cost, and inference time.

Model	Total parameters	Trainable parameters	Inference time (ms/image)	Throughput (images/sec)	Peak GPU memory (MB)
ResNet50	23516228	23516228	6.75	147.97	410.94
ConvNeXT Base	87570564	87570564	10.74	93.04	1576.26
VGG19	139586628	139586628	1.78	559.311	3604.09
EfficientNetV2B0	5863828	5863828	10.07	99.25	1523.48
PoolFormer	11404228	11404228	6.05	165.24	1619.33
ViT	85801732	85801732	5.43	183.85	2095.71
Swin	27522430	27522430	11.82	84.58	1205.12
DeiT	5525188	5525188	5.36	186.24	841.04
H_TL_	131754670	131754670	19.75	50.63	3911.77
H_TF_	100553669	100553669	16.27	61.43	3667.99
H_F_	232701295	232701295	36.07	27.71	5506.83

The final hybrid model (H_F_) exhibits the highest complexity, as reflected in its larger parameter count due to its dual-branch architecture (H_TL_ + H_TF_), attention mechanisms, and feature fusion layers. This translates directly into higher training time and memory requirements than individual CNN and Transformer models. In contrast, simpler architectures such as ResNet50 and VGG19 maintain significantly lower parameter footprints but do not achieve competitive predictive performance.

Despite this increased complexity, the cost remains largely front-loaded during training. At inference, the gap is considerably reduced, with H_F_ showing execution times comparable to other hybrid models and only marginal overhead relative to standalone architectures. This indicates that the added parameters primarily contribute to richer feature representation rather than prohibitive runtime latency.

From a practical standpoint, the higher computational footprint of H_F_ is justified. Training is typically conducted offline on GPU-enabled systems, where increased parameterization is manageable and often expected. More importantly, in the context of brain tumor classification, real-time execution is not a strict requirement, whereas diagnostic accuracy and reliability are critical. Clinical workflows typically allow sufficient processing time for image analysis, and decisions are guided by correctness rather than millisecond-level latency. In this setting, a modest increase in computational cost is a rational trade-off for significantly improved performance and reduced diagnostic errors.

Furthermore, parameter efficiency should be interpreted in context. Models with fewer parameters often lack the capacity to capture complex tumor characteristics, leading to instability under distribution shifts. In contrast, HF leverages its higher capacity to achieve robust generalization, effectively reducing downstream costs associated with misclassification, re-evaluation, and clinical uncertainty. In this sense, the model shifts the burden from unreliable predictions to upfront computation, which is a more practical and clinically aligned compromise.

## 8 Visualization with grad-CAM++

**FIGURE 11:**
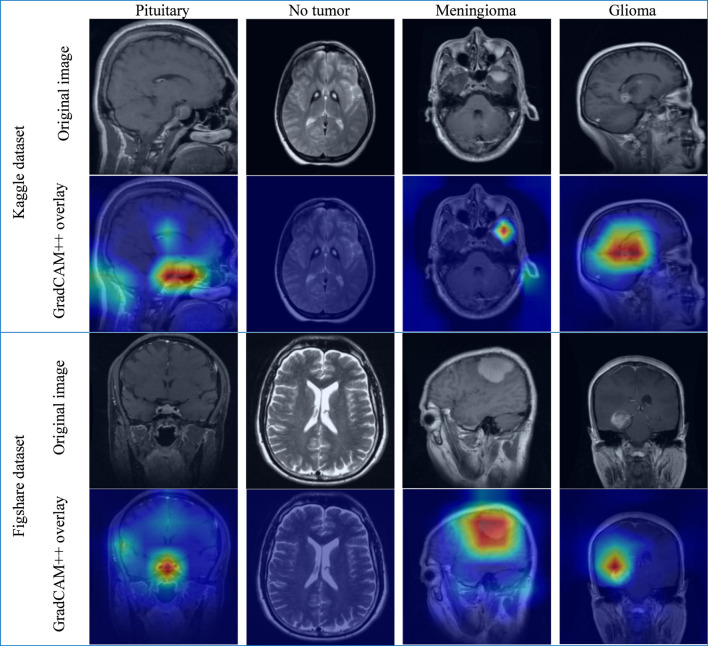
Grad-CAM++ overlay heatmap of four tumour classes on two datasets.

To support interpretability of the model prediction, we applied Grad-CAM++ to generate class-discriminative heatmaps for visual insight into the regions that contribute most to the model’s classification output for four tumor classes. The integration of Grad-CAM++ in brain tumour classification enhances the clinical applicability of deep learning models by providing visual explanations of model decisions. Figure 11 shows four original images representing each tumor class for each dataset are accompanied by the GradCAM++ outputs.

Across both datasets, the Grad-CAM++ maps concentrate activation over the tumor beds for glioma, meningioma, and pituitary cases, with minimal spillover into anatomically irrelevant regions. In the no_tumor examples, saliency remains low or restricted to benign anatomical landmarks (e.g., ventricles or cortical sulci), rather than fabricating spurious “hot spots.” This alignment between heat and lesion locations is consistent across Figshare and Kaggle despite differences in acquisition characteristics, suggesting that the model’s decisions are driven by disease-relevant structure rather than background texture. The overlays also indicate attention to tumor–edema interfaces in glioma and dural attachments in meningioma, while pituitary cases show compact, sellar-centered emphasis that respects the lesion’s small footprint. Any residual off-target glow appears limited and peri-lesional, rather than diffuse or randomly distributed.

## 9 Discussion, clinical relevance, and practical applications

The rationale for the proposed hybrid design is to combine the strengths of both paradigms–CNNs and Transformers. The H_TL_ branch contributes strong local feature extraction capabilities, essential for distinguishing subtle tumor textures and boundaries. The H_TF_ branch contributes global contextual awareness, capturing spatial relationships across distant regions of the brain—crucial for differentiating anatomically similar but spatially distinct tumor types. Their fusion yields a more discriminative and robust feature space, as evidenced by the HF model’s superior performance on both the Kaggle and Figshare datasets. This hybrid architecture, therefore, not only improves accuracy but also enhances generalization under distributional shifts, achieving a balance that neither CNNs nor Transformers achieve independently.

The experimental findings across confusion matrices, evaluation metrics, ROC curves, and statistical tests collectively indicate that the proposed hybrid framework is not only accurate but consistently reliable across datasets. The confusion matrices exhibit strong diagonal dominance for both Kaggle and Figshare, suggesting minimal ambiguity in classification. More importantly, the stability of predictions across variations in acquisition protocols and anatomical diversity indicates that the model is not overly tuned to a specific dataset but captures underlying tumor characteristics. The few observed misclassifications appear to arise from ground-truth variability and noise rather than systematic model bias.

Performance metrics reinforce this observation. Recall and F1-score—arguably the most clinically relevant metrics—reach perfect values on Figshare and remain near saturation on Kaggle, indicating that tumor regions are rarely missed. Crucially, this high sensitivity is not achieved at the expense of precision, which remains consistently high, suggesting that improved detection does not introduce excessive false positives. Given that AUC values are already saturated (∼0.99–1.00), relative differences are more meaningfully reflected through F1, recall, and cross-dataset consistency, where the final hybrid model (H_F_) demonstrates the most stable behavior.

This stability can be traced back to the architectural design. CNN-based models exhibit sensitivity to dataset heterogeneity, while Transformer-based models show minor drift due to weaker local inductive biases. The hybrid framework mitigates both limitations by integrating local spatial detail with global contextual reasoning. As a result, boundary delineation improves and subtle tumor regions are detected more consistently, reducing both false negatives and spurious activations.

The ROC curves further support this behavior, with H_F_ consistently approaching the top-left boundary across both datasets. This indicates strong class separability and stable confidence across varying thresholds, which is particularly relevant in clinical settings where operating points may shift depending on diagnostic priorities.

Statistical analysis strengthens these observations. The Friedman test confirms that performance differences are not due to chance, and H_F_ consistently attains the top rank across datasets. Holm’s *post hoc* analysis shows significant improvements over most baseline models. Although H_TL_ and H_TF_ are not significantly different, neither consistently achieves top performance across datasets. H_F_, by contrast, remains stable under distribution shifts, suggesting that its advantage lies not in isolated gains but in sustained reliability.

The CD diagrams further reveal that this superiority is structurally grounded. Performance improves progressively from single models to intra-family hybrids and finally to the fused architecture, indicating that gains arise from a systematic reduction in representational limitations rather than from incidental improvements. The tighter clustering observed in Figshare suggests dataset saturation, whereas the clearer separation in Kaggle provides stronger evidence of real-world robustness.

Calibration analysis adds an important layer of validation. HF produces well-calibrated probability estimates across both datasets, with only minor deviations under more challenging conditions (Kaggle). The near-perfect calibration observed on Figshare likely reflects lower data complexity and should be interpreted as an upper-bound scenario. In contrast, the controlled deviation on Kaggle indicates appropriate uncertainty handling and avoids overconfident predictions. This distinction is critical, as reliable confidence estimation directly supports risk-aware clinical decision-making.

The TOPSIS analysis confirms that HF is not merely superior in isolated metrics but achieves the most balanced overall performance. Aggregating multiple evaluation criteria highlights that HF avoids the common trade-offs seen in competing models. This balance is particularly important in brain tumor diagnosis, where both missed detections and false alarms have serious clinical implications. The consistent top ranking across datasets reinforces that the hybrid design delivers not just higher accuracy, but more dependable and clinically aligned performance.

The Grad-CAM++ overlays provide a face-valid explanation: predictions arise from features that a radiologist would also look at. In glioma, attention spreads along infiltrative margins and FLAIR-like hyperintense rims, a pattern consistent with the biology of diffuse tumors; in meningioma, heat concentrates along extra-axial masses with apparent dural tails; in pituitary, the emphasis is tightly centered in the sella, reflecting the small, well-circumscribed lesion profile. For no-tumor images, the absence of pathologic saliency argues against shortcut learning from background artifacts. The cross-dataset stability of these patterns is notable: the same classes exhibit similar saliency footprints on Figshare and Kaggle, despite differences in contrast, resolution, and noise. Limitations are visible in a minority of overlays—edge attenuation in larger gliomas (partial under-attention to distal edema) and mild peri-lesional spill in meningioma—but these are contained and do not suggest systematic failure. Overall, the maps indicate that the hybrid model’s discriminative cues are lesion-centric and anatomically plausible, not merely texture-based or scanner-specific.

The clinical relevance of the proposed hybrid model extends beyond numerical accuracy. In brain tumor diagnosis, the primary clinical concern is not simply whether a model can classify or segment correctly under ideal conditions, but whether it can do so reliably in the unpredictable and heterogeneous conditions of real-world healthcare. The observation of our model’s near-perfect Recall values indicates that it rarely misses tumor tissue, which is crucial because false negatives, especially in gliomas and infiltrative tumors, can lead to underestimation of tumor extent, incomplete surgical resection, or delays in treatment planning. Even a small, undetected lesion may lead to disease progression or recurrence, making high sensitivity arguably the most clinically important metric in this domain.

Moreover, the model sustains high precision, which implies it avoids excessive over-segmentation of healthy tissue. From a clinical standpoint, this is just as important in surgical and radiotherapy planning. Overestimating tumor volume can result in unnecessarily aggressive resection, affecting functional areas of the brain and causing avoidable neurological deficits. In radiotherapy, an exaggerated target region exposes healthy tissue to harmful radiation, increasing long-term cognitive and motor complications. The hybrid model’s ability to balance both precision and sensitivity helps construct tumor contours that are reliable for planning interventions where millimetric accuracy is vital.

In radiology workflows, this model can serve as a second reader: not to replace the radiologist but assist in early screening, preliminary reporting, and consistency checks. For junior radiologists or overburdened clinical setups, it reduces the cognitive load by automatically localizing suspicious regions. In tertiary hospitals, it can support neuronavigation systems, provide volumetric tumor measurement, tracking changes across time, and assist in radiogenomic correlation studies. Its generalization across datasets suggests it can adapt to differences in MRI scanners (1.5T vs 3T), protocols (T1, T2-FLAIR, contrast-enhanced), and patient populations, a prerequisite for widespread clinical integration.

The practical implications also extend to interdisciplinary use. Neurosurgeons can use segmentation outputs intraoperatively via augmented reality overlays or preoperative planning software. Oncologists can rely on model-generated volumetric measurements for evaluating chemotherapy or radiotherapy response, thereby reducing subjective bias. Medical physicists can incorporate segmentation maps to automate radiotherapy contouring, cutting down time spent on manual delineation.

In low-resource settings or rural hospitals where expert neuroradiologists are unavailable, such a model gives clinicians decision support that approximates expert-level segmentation performance. Reducing variability between observers it promotes diagnostic uniformity across institutions, which is essential for multi-centre treatment planning and clinical trials.

In brain tumor diagnosis and treatment planning, interpretability is critical—particularly when automated segmentation influences surgical decisions or radiotherapy targeting. The Grad-CAM++ visualizations provide clinicians with a direct way to verify whether the model bases its predictions on clinically meaningful regions. In glioma cases, the heatmaps consistently highlight peri-lesional areas and infiltrative borders, which aligns with the clinical priority of detecting microscopic tumor spread. For meningiomas, attention is focused on extra-axial masses and dural attachments, reinforcing confidence in both tumor localization and boundary identification. In pituitary tumors, the saliency remains compact within the sella turcica, avoiding misinterpretation of adjacent structures. Equally important, in no-tumor cases, the absence of irrelevant activation reassures clinicians that the model is not generating false alarms that could lead to unnecessary imaging or intervention.

This level of transparency makes the model easier to trust and adopt. Radiologists can quickly cross-check model attention with standard imaging cues, while multidisciplinary tumor boards can use the heatmaps to justify or question AI-generated outputs when deciding on resection margins or treatment volumes. When attention maps diverge from anatomical or pathological expectations, clinicians should review the prediction rather than accept it at face value. In this way, Grad-CAM++ transforms a black-box model into an interpretable system with a traceable rationale behind each decision.

From a practical standpoint, these heatmaps can be integrated at multiple stages of clinical workflow. During triage or second-read scenarios, especially in high-volume or resource-constrained settings, automatic saliency maps can flag suspicious regions for rapid assessment. In refinement and editing, pairing heatmaps with segmentation outputs allows clinicians to focus manual corrections on areas where the model shows uncertainty—such as regions that are activated but not fully segmented. For reporting and quality assurance, exporting the heatmap alongside the final mask creates a lightweight interpretability layer that can be reviewed in multidisciplinary meetings or stored for longitudinal comparison.

Effective deployment requires that the heatmaps be co-registered with the original MRI sequence, normalized for consistency, and displayed with adjustable opacity within PACS or radiotherapy planning systems. Archiving these overlays alongside the segmentation outputs also supports retrospective auditing when imaging findings and patient outcomes diverge.

## 10 Conclusions, limitations and future scope

This study proposes a transfer learning–Transformer hybrid framework for brain tumor segmentation and demonstrated its effectiveness across two heterogeneous MRI datasets. The final hybrid model (H_F_), which integrates convolution-based (H_TL_) and Transformer-based (H_TF_) hybrids, consistently outperformed individual backbone networks and their intermediate combinations. Its superiority was not limited to accuracy but extended to clinically meaningful metrics such as Recall and F1-score, where missed tumor regions carry far greater consequences than marginal declines in precision. Statistical validation using Friedman’s rank-based test and Holm’s *post hoc* test confirmed that HF maintains its top rank across datasets and is significantly different from most baseline architectures. In sum, the final hybrid model outperforms other architectures on standard metrics. It demonstrates traits that matter in real clinical practice—consistent sensitivity across datasets, resistance to distribution shifts, balanced precision to prevent overtreatment, and statistical robustness validated through rank-based and *post hoc* testing. These attributes justify its consideration not merely as a research contribution but as a viable assistive tool in radiology and neurosurgical workflows.

Interpretability analysis using Grad-CAM++ further strengthened the framework’s reliability. The saliency maps indicated that the model’s predictions were guided by anatomically and pathologically relevant regions, such as tumor core, enhancing margins, peri-lesional edema, and in the case of pituitary tumors, the sella turcica. The Grad-CAM++ evidence indicates that model decisions are anchored in the pathology itself rather than confounds or background textures. The overlays are consistent across datasets and tumor classes, disciplined in no-tumor scenarios, and only modestly affected by edge cases. When used alongside the segmentation masks, these maps enhance clinical confidence, guide targeted corrections, and create an audit trail that makes the system safer to deploy. Together, the results suggest that the hybrid model and its interpretability pipeline can serve as a reliable decision-support tool across clinical workflows, ranging from early detection to treatment planning and follow-up evaluation.

Despite these strengths, several limitations warrant consideration. The datasets used, although diverse, still represent curated imaging conditions; real-world hospital data often include artifacts, low-field MRI scans, motion distortion, and incomplete modalities, which the model has not yet encountered. The study focused on multi-class tumor classification but did not incorporate tumor grading or radiogenomic mapping, which are critical for prognosis. Additionally, while Grad-CAM++ provides useful visual insights into the model’s decision-making process, it does not fully capture the complex feature interactions within deep attention layers, particularly in hybrid architectures. Its reliability may also diminish near class boundaries, especially in cases of highly infiltrative tumors where distinctions between tumor and surrounding tissue are less well-defined. Moreover, the current explainability analysis remains qualitative. Although the visualizations suggest that the model attends to clinically relevant tumor regions, they serve primarily as a plausibility check rather than a rigorous localization assessment. The absence of standardized pixel-level annotations across all samples prevents the use of quantitative evaluation metrics such as Dice coefficient, Intersection over Union, or pointing-game accuracy. As a result, the interpretability findings should be viewed as supportive rather than definitive.

In addition, the increased computational complexity of the hybrid architecture may pose challenges for deployment in low-resource clinical settings. Future work will focus on addressing these limitations through quantitative validation of localization using annotated tumor masks, expert-driven evaluation, and exploration of more computationally efficient model variants.

Future work should extend this framework to multi-institutional MRI data including variations in scanner strength, imaging protocols, and patient demographics to test genuine clinical generalizability. Incorporating 3D volumetric models, multimodal MRI sequences (T1, T2, FLAIR, contrast-enhanced), and longitudinal follow-up scans could enhance temporal stability and capture progression or treatment response. Integration with radiogenomics and survival prediction models could transform this system from a diagnostic tool into a comprehensive prognostic aid. Further research into lightweight hybrid variants, uncertainty quantification, and federated learning could enhance the model’s adaptability to real-time, privacy-preserving hospital environments.

## Data Availability

The datasets used in the experiment are publicly available at https://github.com/Kirti-Pant/ConvNeXT-Base-for-Brain-Tumor-Classification--Data.git.
